# Functional impairment as a proxy measure indicating high rates of trauma exposure, post-migration living difficulties, common mental disorders, and poor health amongst Rohingya refugees in Malaysia

**DOI:** 10.1038/s41398-019-0537-z

**Published:** 2019-09-02

**Authors:** Alvin Kuowei Tay, Susan Rees, Mohammed Abdul Awal Miah, Sanjida Khan, Mohammad Badrudduza, Karen Morgan, Darlina Fadil Azim, Susheela Balasundaram, Derrick Silove

**Affiliations:** 1 Faculty of Medicine, School of Psychiatry, University of New South Wales, Sydney, NSW 2052 Australia; 2 Department of Psychology, Jagannath University, Dhaka, Bangladesh; 3 School of Medicine, Perdana University-Royal College of Surgeons in Ireland (PU-RCSI), Selangor, Malaysia; 4 Health Unit, United Nations High Commissioner for Refugees (UNHCR), Kuala Lumpur, Malaysia

**Keywords:** Human behaviour, Scientific community

## Abstract

A major challenge in the refugee field is to ensure that scarce mental health resources are directed to those in greatest need. Based on data from an epidemiological survey of 959 adult Rohingya refugees in Malaysia (response rate: 83%), we examine whether a brief screening instrument of functional impairment, the WHO Disability Assessment Schedule (WHODAS), prove useful as a proxy measure to identify refugees who typically attend community mental health services. Based on estimates of mental disorder requiring interventions from analyses of epidemiological studies conducted worldwide, we selected a WHODAS cutoff that identified the top one-fifth of refugees according to severity of functional impairment, the remainder being distributed to moderate and lower impairment groupings, respectively. Compared to the lower impairment grouping, the severe impairment category comprised more boat arrivals (AOR: 5.96 [95% CI 1.34–26.43); stateless persons (A20·11 [95% CI 7.14–10); those with high exposure to pre-migration traumas (AOR: 4.76 [95% CI 1.64–13.73), peri-migration stressors (AOR: 1.26 [95% CI 1.14–1.39]) and postmigration living difficulties (AOR: 1.43 [95% CI 1.32–1.55); persons with single (AOR: 7.48 [95% CI 4.25–13.17]) and comorbid (AOR: 13.54 [95% CI 6.22–29.45]) common mental disorders; and those reporting poorer general health (AOR: 2.23 [95% CI 1–5.02]). In addition, half of the severe impairment grouping (50.6%) expressed suicidal ideas compared to one in six (16.2 percent) of the lower impairment grouping (OR: 2.39 [95% CI 1.94–2.93]). Differences between the severe and moderate impairment groups were similar but less extreme. In settings where large-scale epidemiological studies are not feasible, the WHODAS may serve as readily administered and brief public health screening tool that assists in stratifying the population according to urgency of mental health needs.

## Introduction

Recent United Nations reports have documented the extensive human rights violations committed over several decades against the Rohingya minority of Myanmar^1,2^. Repeated episodes of violence and persecution committed by the Burmese military have forced successive waves of Rohingya—in total over 1.1 million since the 1980s—to flee their homeland in Rakhine state to surrounding Southeast Asian countries where they now constitute the largest population of refugees in the region (see Supplementary File 1 for the history of persecution of this group)^1^.

Identifying refugees in most urgent need of mental health and psychosocial (MHPSS) assistance presents a major challenge for planners and service providers when dealing with such large populations of displaced persons. In the present study, based on a large sample of Rohingya relocated to Malaysia, we examine whether a brief screening measure of functional impairment, the WHODAS, can identify refugees in greatest need of MHPSS services. Based on global estimates derived from two reviews of the literature^3,4^, between 10 and 15% of refugee populations require assistance for mental health problems. For the purposes of illustrating the potential use of the WHODAS as a screening measure, we therefore identify those in the highest 20% in the expectation that in real life health systems, only a half of these (that is, 10% of the total population) will seek or have access to services.

Existing methods for identifying mental health needs amongst large refugee populations tend to be limited in their utility because of their lack of specificity in identifying persons in urgent need of MHPSS care. Screening questionnaires based on mental health symptoms applied to refugees suggest that one in three refugees require attention, a task that far exceeds the capacity of existing services, for example, those available to the Rohingya in various sites in Southeast Asia^3^. As indicated, in many situations of refugee concentration, there is a dearth of studies indicating population-wide MHPSS needs. One extant study based on a small sample of Rohingya refugees (*n* = 140) living in the humanitarian camps in Bangladesh found high rates of depression (89%) and posttraumatic stress disorder (PTSD) (36%), figures that provide little guidance in identifying those in most urgent need of intervention.

A further consideration is that refugees attending mental health services typically report experiencing a combination of CMDs (often in comorbid form) and a range of past and ongoing psychosocial stressors, including pre-migration traumatic events (TEs), peri-migration stressors experienced during flight from the home country, and post-migration living difficulties (PMLDs)^5,6^. Assessing the full range of these experiences requires detailed and sensitive interviewing. Many also report physical health problems, adding to the complexity of assessments^7^. If a measure of functional impairment can provide a proxy index identifying refugees who exhibit this complex combination of problems, this would greatly aid the process of ensuring that scarce mental health services are used most efficiently in addressing those in greatest need of assistance.

In the present study, we examine the novel application of a brief measure of functional impairment, the WHODAS, to assess whether it may serve as a proxy indicator of the range of MHPSS problems commonly manifested by refugees attending mental health services. In past studies in the field, measures of functional impairment have been used to indicate the practical outcomes of MHPSS problems^8,9^. Here we reverse the role by testing the use of the WHODAS as a possible proxy indicator of underlying MHPSS problems. We chose the WHODAS because it is a brief and widely applied measure of functional impairment with extant studies demonstrating^8,10–12^ sound psychometric properties.

Specifically, we examine whether stratification according to the WHODAS of refugees participating in a representative epidemiological study corresponds with a hierarchy of severity of MHPSS problems typical of patients attending mental health services. We predict that refugees assigned to the top 10% of scores on the WHODAS will report the highest prevalence of single and comorbid CMDs and physical ill-health as well as the most extreme exposure to pre-migration traumatic events and peri- and postmigration stressors.

## Materials

### Subjects and methods

#### Base population and sampling

According to estimates by community leaders, non-government organizations, and the UNHCR in Malaysia, 75% of the Rohingya community reside in and around the two major cities of Kuala Lumpur in Selangor state and Ipoh in Perak state. As a distinct group, the community lives in dense clusters in these geographical localities. Applying a cluster, probability proportional-to-size sampling framework, we identified 18 villages in Selangor and 31 in Perak (see Supplementary File 1 for full details). The survey team commenced at a fixed point and then identified all consecutive households along the street from one end to the other. We mapped and recorded all eligible persons in each dwelling. Eligible persons were self-identified Rohingya originating from Myanmar or the offspring of at least one Rohingya parent. Based on our power analyses (90% power; alpha = 0.05, two-sided test), our minimum sample size was 600 persons (approximately 300 in each of the two states). Of the 1156 adults approached, 197 (17%) declined interview (all because of time commitments given their work schedules) the remainder consented to be interviewed, yielding a response rate of 83%.

Ethical permission for the study was provided by the University of New South Wales Human Research Ethics Committee, Australia; and Perdana University-Royal College of Surgeons in Ireland School of Medicine, Malaysia, Institutional Review Board.

#### Survey instruments

We used the Refugee Mental Health Assessment Package (R-MHAP)^13^ to assess trauma events (TEs), peri-migration stressors, postmigration living difficulties (PMLDs) and four common mental disorders (CMDs) relevant to the refugee experience (listed hereunder). Fuller details are provided in Supplementary File 2.

### Mental health indices

We assessed current diagnoses of four CMDs known to be common amongst refugees, that is, posttraumatic stress disorder (PTSD), major depressive disorder (MDD), generalized anxiety disorder (GAD) and persistent complex bereavement disorder (PCBD)^13^ based on the Diagnostic and Statistical Manual, 5th edition (DSM5). Given that interviews were conducted by trained non-professional field workers, we refer to diagnoses as “probable”. Suicidal ideation was assessed using an item on the depression screen: “how often have you thought about taking your own life in the last two weeks?”. Persons identified as a suicide risk (who responded “yes”) were referred to UNHCR affiliated health partners in Malaysia for a comprehensive assessment and where necessary, treatment.

We used the widely applied 12-item Short Form Health Survey (SF-12) to assess reports of general physical ill-health^14^. The item has yielded high levels of reliability and validity in comparison to more extensive assessments of physical health in past studies^14^.

The WHO Disability Assessment Schedule-Short Form (WHODAS 2.0)^15^ has been widely used in epidemiological surveys worldwide, yielding sound psychometric properties across cultures and contexts. The WHODAS was translated into the Rohingya language and adapted to the culture of Rohingya refugees living in Malaysia. For indicative purposes, we categorized persons into three impairment groups, that is, high (approximately top twenty percent), moderate (next one fifth), and lower (bottom three fifths). The closest rounded score on the WHODAS was 32 which identified 166 persons (17.3% of the sample) who then formed the indicative high impairment group; the moderate impairment group (WHODAS score of 19–31 inclusive) included 220 persons (22.9%); and the lower impairment group (WHODAS score 0–18) comprised 573 persons (59.8%).

### Psychosocial adversity

Lifetime exposure to pre-migration traumatic events (TEs) was measured by the R-MHAP module adapted to the context and history of the Rohingya. Items included exposure to war, torture, persecution, rape, murders, physical injuries, imprisonment, witnessing atrocities, and witnessing deaths of family members. The TE count ranged from 0 to 12. To ensure roughly proportional representation in each category based on an examination of the distribution of scores, we derived three ordinal categories (0–10, 11–20, 20 counts or more).

Peri-migration stressors included extortion by people smugglers, starvation, physical assault, sexual abuse, and witnessing deaths or suicide of others. We applied the same binary response scale (exposed or not) as for the TE count. Peri-migration stressors (range 0–13) were ordered according to three ordinal categories: 0–5, 6–10, 11 counts or more.

An adapted inventory of post-migration living difficulties (PMLDs)^16^ was used to assess common stressors confronted by the Rohingya communities in Malaysia. We used binary categories (0 = not a problem or a bit of a problem; 1 = a moderately or a very serious problem), generating a total PMLD count (range 1–25). We derived three ordinal categories: 0–10, 11–20, 20 counts or more.

All survey measures were translated into the Rohingya language. Interviews were conducted verbally in Ruáingga by field workers employed from the local Rohingya communities and responses were recorded on electronic tablet devices. The field team completed two weeks of training covering, inter alia, basic interviewing techniques (e.g. paraphrasing, clarifying, emphatic listening), refugee mental health, cultural sensitivity, research ethics, and risk assessment and management. The interviews took on average 45–60 min.

#### Statistical analysis

We calculated sample size on expected odds ratios of major predictors of functional impairment (3.4 or greater). After generating frequencies and prevalence data for all variables, we conducted bivariate analyses to examine associations of explanatory variables with our three-tier functional impairment typology. Explanatory variables included social and demographic characteristics, length of residency in Malaysia, residency status, pre-migration TEs, peri-migration stressors (including travel mode), PMLDs, CMD status (one disorder, or two or more disorders), severity of suicidal ideation, and self-rated physical health (poor/fair vs good/excellent health) (see Supplementary File 2).

Multinomial logistic regression analysis was then used to characterize refugees assigned to the three functional groups, excluding variables (education and length of residency) which were not significant in bivariate analyses. Collinearity (VIF > 3·3) amongst variables required that we conducted three separate regression models: Regression 1. Demographic and migration variables (age, gender, marital status, residency status, travel mode); Regression 2. Trauma and stressor variables: TEs, peri-migration stressors and PMLDs; Regression 3. CMD status and physical health. Suicidality was examined as a separate analysis given that the index formed part of the measure of major depression. We report odds ratios (ORs) and adjusted odds ratios (AORs) with 95% confidence intervals (CIs) in each instance. Missing data were excluded from the analyses (see Supplementary File 2 for details).

## Results

Table 1 reports the sociodemographic characteristics of the analytic sample of 959 adults with a mean age of 28,3 years (sd = 9,03), comprising 743 men (77.5%). Refugees had resided for an average 47 months (~4four years) in Malaysia. The majority had arrived in Malaysia by boat (*n* = 835, 87.1%) and received humanitarian protection status by UNHCR Malaysia (*n* = 687, 71.6%). A quarter had completed primary school (*n* = 261, 27.2%) and over a half were employed (*n* = 632, 65.9%). Close to half of all adults were married (*n* = 466, 48.6%).

**Table 1:** Social and demographic characteristics of the sample (*n* = 959)

Sociodemographic variables	*N*	%/sd
*Age*		
18–30	644	67.2%
31–40	175	18.2%
41 and above	140	14.6%
Mean age (in years)	28.3	9.03
average length of time (in months) in Malaysia	47	43.68
*Gender* ^a^ ^g^		
Female	171	17.8%
Male	743	77.5%
Total/combined	914	95.3%
*Employment* ^bg^		
Unemployed	120	12.5%
Employed/domestic duties	632	65.9%
Total/combined	752	78.4%
*Educational attainment* ^cg^		
Illiterate/no education	538	56.1%
Completed primary education	261	27.2%
Total/combined	799	83.3%
*Marital status* ^dg^		
Single/unmarried	360	37.5%
Married/widowed	466	48.6%
Total/combined	826	86.1%
*Residency status* ^eg^		
UNHCR protection status	687	71.6%
Stateless	183	19.1%
Total/combined	870	90.7%
*Mode of travel to Malaysia* ^fg^		
By boat	835	87.1%
By car or other means	73	7.6%
Total/combined Total/combined	908	94.7%

^a^41 missing

^b^207 missing

^c^160 missing

^d^133 missing

^e^89 missing

^f^51 missing

^g^missing data

Descriptive data for TEs, peri-migration stressors, and PMLDs for the sample as a whole are reported in Supplementary File 2). In relation to TEs, the most widely endorsed items were torture (*n* = 775, 81%), witnessing rape and other forms of sexual violence (*n* = 771, 80%), shortage of food and water (*n* = 660, 69%), and witnessing murders of friends and family members (*n* = 611, 64%). In relation to peri-migration stressors, the majority of respondents reported being confined in overcrowded spaces with others for weeks (*n* = 923, 96.2%), experiencing financial extortion by people smugglers (*n* = 915, 95.4%), hiding to escape police or other authorities (*n* = 872, 90.9%),and shortage of food and water during long journeys (*n* = 828, 86.3%). The PMLDs identified as either a serious or a very serious problem included: poverty (*n* = 864, 90.1%); lack of aid from NGOs (*n* = 932, 97.2%); poor access to healthcare (*n* = 788, 82.2%); and inadequate shelter (*n* = 747, 77.9%).

Table 2 reports the prevalence of the four probable CMD diagnoses. One third (306, 32%) met criteria for PTSD, 115 (12%) for MDD, 83 (9%) for GAD, and 70 (7%) for PCBD. Of those with a CMD, a third (329, 34.3%) had a single disorder, and 617 (64.3%) had two or more disorders. Almost a quarter (265, 27.7%) rated their current physical health as fair or poor. Table 3 reports the distributions of key variables by severity of functional impairment.

**Table 2:** Estimate prevalence of comorbid mental disorders based on DSM-5 criteria with severe functional impairment in a community sample of Rohingya refugees living in Malaysia (*n* = 959)

Probable diagnoses of comorbid mental disorders	Any disorder		Posttraumatic stress disorder		Generalized anxiety disorder		Major depressive disorder		Persistent complex bereavement disorder	
	*N*	%	*N*	%	*N*	%	*N*	%	*N*	%
Estimate prevalence based on DSM-5 criteria + severe impairment	414	43.2%	306	31.9%	83	8.7%	115	12%	70	7.3%

Operational criteria for deriving probable diagnoses using severe rating for functional impairment on the R-MHAP for PTSD, GAD, MDD, and PCBD. In addition, we specified additional criteria for PTSD in which symptom duration must exceed 3 months; for PCBD, symptoms must be experienced every day since onset of loss in the last 12 months with severe impairment

**Table 3:** Distributions of sociodemographic variables, pre-migration, peri-migration, post-migration ∞ trauma-related and psychosocial risk factors, mental disorders, and physical impairment by severity of functional impairment (*n* = 959) ±

Sociodemographic variables	Low (*n* = 573)		Moderate (*n* = 220)		Severe (*n* = 166)		X2 tests
	*N*	%	*N*	%	*N*	%	
*Age*							
18—30	370	64.6	148	67.3	126	75.9	
31—40	104	18.2	42	19.1	29	17.5	
41 and above	99	17.3	30	13.6	11	6.6	*P* = 0.021
*Gender* ^a^							
Female	136	25.6	26	11.9	9	5.3	
Male	395	74.4	192	88.1	156	94	*P* < ·001
*Employment* ^a^							
Unemployed	78	17.8	34	21	8	5.3	
Employed/domestic duties	360	82.2	128	79	144	86.7	*P* < 0.001
*Educational attainment* ^a^							
Illiterate/no education	307	66.2	134	69.4	97	68.3	
Completed primary education	157	33.8	59	30.6	45	31.7	*P* = 0.692
*Marital status* ^*a*^							
Single/unmarried	184	38.7	90	46·6	86	54·4	
Married/widowed	291	61.3	103	53.4	72	45.6	*P* < 0.001
*Residency status* ^a^							
Held UNHCR refugee status^b^	382	72.8	174	82.5	131	97.8	
Stateless (without UNCHR protection)^c^	143	27.2	37	17.5	3	2.2	*P* < 0.001
*Exposure to pre-migration TEs*							
0–10 counts	273	47·6	70	31·8	67	40.4	
11–20 counts	264	46.1	124	56.4	61	36.8	
21 counts or more	36	6.3	26	11.8	38	22.9	*P* < 0.001
*Exposure to peri-migration stressors*							
0–5 counts	157	27.4	34	15.5	11	6.6	
6–10 counts	333	58.1	133	60.5	77	46.4	
11 counts or more	83	14.5	53	24.1	78	47	*P* < 0.001
*Mode of travel to Malaysia* ^a^							
By boat	469	89.2	203	93.6	163	98.8	
By car or other means	57	10.8	14	6.5	2	1.2	*P* < 0.001
*Post-migration living difficulties* ^d^							
0–10 counts	206	37.3	36	16.7	5	3.1	
11–20 counts	302	54.7	165	76.7	122	74.4	
21 counts or more	44	8	14	6.5	37	22.6	*P* < 0.001
*Overall self-rated physical health*							
Excellent/very good	168	70.6	59	26.8	38	22.9	
Fair/poor	404	29.4	161	73.2	128	77.1	*P* < 0.001
*Mental disorders*							
No disorder	397	69.3	104	47.3	44	26.5	
One disorder	138	24.1	76	34.6	82	49.4	
Two or more disorders	38	6.6	40	18.2	40	24.1	*P* < 0.001

^a^missing data on key demographic variables were excluded from the analysis

^b^Participants were recognized as genuine refugees by UNHCR following Refugee Status Determination (RSD)

^c^Participants did not hold UNHCR protection status at the time of the survey

^d^Post-migration living difficulties were endorsed at moderate or severe levels

### Bivariate analyses

Table 4 reports bivariate analyses comparing severe versus lower functional impairment; moderate versus lower impairment; and severe versus moderate impairment categories respectively. For brevity, we report here only differences between the severe and lower impairment categories, the criterion used for inclusion of variables in subsequent multinomial regression analyses. Compared with the lower impairment category, more members of the severe impairment category were young, male, single, employed, boat arrivals, and stateless persons. In addition, the severe impairment group reported greater exposure to TEs (21 counts or more), peri-migration stressors (11 counts or more), and PMLDs (11–20, 21 counts or more). The severe impairment group also reported higher rates of single and multiple CMDs; and of poor general health (*P* < 0.05 for all *X*^2^ tests).

**Table 4:** Univariate multinomial logistic regressions examining associations of sociodemographic, trauma-related, psychosocial, mental and physical predictors of severity of functional impairment in a community sample of Rohingya refugees living in Malaysia (*n* = 959)

Reference group: low impairment (*n* = 573)	Moderate impairment (*n* = 220)			Severe impairment (*n* = 166)		
Sociodemographic characteristics	Odds Ratios	95% Confidence Interval	*P*	Odds Ratios	95% Confidence Interval	*P*
Age Base cat: 18–30 years	0.85	0.73–0.98	0.029	0.70	0.58–0.86	<0.001
Male sex Base cat: female	2.54	1.62–4	<0.001	5.97	2.96–12	<0.001
Being employed Base cat: unemployed	0.97	0.61–1·54	0.90	5.15	2.41–10.96	<0.001
Completed primary or secondary education Base cat: illiterate/no education	0.84	0.61–1.17	0.299	0.89	0.63–1.28	0.544
Being married Base cat: single	0.86	0.66–1.13	0.28	0.64	0.47–0.86	0.004
Residency status (being stateless) Base cat: UNHCR protection status	1.75	1.18–2.63	0.006	16·67	5–50	<0.001
Exposure to pre-migration Traumatic Events (TEs) (mean score)	3.27	1·70–6·29	<0.001	3·4	1·66–7·03	<0.001
Peri-migration stressors (base cat: 0 count)	1.11	1.05–1.16	<0.001	1·38	1.27–1.49	<0.001
Traveled to Malaysia by boat Base cat: traveled by land transport	1.76	0.96–3·23	0.067	9.90	2.39–41	0.002
Post-migration living difficulties (PMLDs)						
Exposure to post-migration living difficulties endorsed at moderate and severe levels (mean score)	1.08	1.05–1.12	<0.001	1·25	1.20–1.3	<0.001
Overall self-rated physical health (base cat: very good/excellent)						
Poor/fair	2.16	1·17–3·98	0.337	2.16	1.17–3.98	0.013
Mental disorders (base cat: no disorder)						
One disorder	2.1	1.48–2.99	<0.001	4.02	2.45–6.58	<0.001
Two or more disorders	5.36	3.54–8.11	<0.001	9.50	5·.52–16.34	<0.001

### Multinomial logistic regression analyses

Table 5 reports the findings of the multivariate analyses.

**Table 5:** Multivariate multinomial logistic regressions^#^ examining associations of sociodemographic, trauma-related, psychosocial, mental and physical health predictors of functional impairment by levels of severity in a community sample of Rohingya refugees living in Malaysia (*n* = 959)

Reference group: low impairment (*n* = 573)	Moderate impairment (*n* = 220)			Severe impairment (*n* = 166)		
Sociodemographic characteristics	Adjusted odds ratios	95% confidence interval	*P*	Adjusted odds ratios	95% confidence interval	*P*
Age Base cat: 18–30 years	0.87	0.65–1.15	0.319	1.03	0.73–1.44	0.874
Gender Base cat: female	2.27	0.82–6.28	0.116	2.22	0.22–5.44	0.982
Being employed Base cat: unemployed	0.87	0.51–0.54	0.90	4.15	1·41–8.96	<0.001
Being married Base cat: single	1.17	0.79–1.73	0.424	0.67	0.42–1.06	0.085
Residency status (being stateless) Base cat: held UNHCR protection status	1.75	1.06–2.94	0.028	20.11	7.14–10	<0.001
Exposure to pre-migration Traumatic Events (TEs) (mean score)	3.19	1.22–8.34	0.018	4.76	1.64–13.73	0.004
Peri-migration stressors (base cat: 0 count)^a^	1.05	0.98–1.12	0.188	1.26	1.14–1.39	<0.001
Traveled to Malaysia by boat Base cat: traveled by land transport	1.23	0.59–2.53	0.581	5.96	1.34–26.43	0.019
Post-migration living difficulties (PMLDs)						
Exposure to post-migration living difficulties endorsed at moderate and severe levels (mean score)	1.1	1.04–1.15	<0.001	1.43	1.32–1.55	<0.001
Overall self-rated physical health (base cat: very good/excellent)^a^						
Poor/fair	1.90	0.91–3.96	0.088	2.23	1–5.02	0.05
Mental disorders (base cat: no disorder)^a^						
One disorder	1.65	1.04–2.61	0.034	7.48	4.25–13.17	<0.001
Two or more disorders	4.03	1.97–8.27	<0.001	13.54	6.22–29.45	<0.001

^a^Given high multi-collinearity between mental disorders, general health, pre- and per-migration exposure and PMLDs, these variables were entered into separate multivariate logistic regressions; education was excluded because of high multi-collinearity

#### Severe versus lower impairment categories

Compared with the lower impairment category, the severe impairment category comprised more boat arrivals (AOR: 5.96 [95% CI 1.34–26.43) and stateless persons (AOR: 20.11 [95% CI 7.14–10). The severe impairment category also reported higher rates of pre-migration TEs (AOR: 4.76 [95% CI 1.64–13.73), peri-migration stressors (AOR: 1·26 [95% CI 1.14–1.39]) and PMLDs (AOR: 1.43 [95% CI 1.32–1.55). The severe impairment group reported higher rates of single (AOR: 7·48 [95% CI 4.25–13.17]) and in particular, comorbid (AOR: 13.54 [95% CI 6.22–29.45]) CMDs, as well as poorer general health (AOR: 2.23 [95% CI 1–5.02]).

#### Moderate versus lower impairment categories

Compared with the lower impairment group, more members of moderate impairment category were stateless (AOR: 1.75 [95% CI 1.06–2.94). They also experienced greater exposure to both pre-migration TEs (AOR: 3.19 [95% CI 1.22–8.34]) and PMLDs (AOR: 1.1 [95% CI 1.04–1.15]); and had a higher prevalence of single (AOR: 1.65 [95% CI 1.04–2.61]) and comorbid (AOR: 4.03 [95% CI 1.97–8.27]) CMDs. There was no difference between the moderate and lower impairment groups in physical health status.

#### Severe versus moderate impairment categories

Compared with the moderate impairment category, the severe impairment category comprised more men (AOR: 2.51 [95% CI 1–6.28]), persons who were employed (AOR: 3.99 [95% CI 1.63–9.76]) and those who were stateless (AOR: 10 [95% CI 2.86–3.33]). The severe impairment category also reported higher rates of both peri-migration stressors (AOR: 1.16 [95% CI 1.04–1.30]) and PMLDs (AOR: 1.37 [95% CI 1.24–1.51]). They recorded more single (AOR: 4.30 [95% CI 2.21–8.35]) and comorbid (AOR: 3.19 [95% CI 1.49–6.81]) CMDs. There was no difference in TEs and physical health status between the two impairment groups.

There was a clear gradient in relation to the item assessing severity of suicidal ideation. Half (50.6%) of those in the severe impairment group, a quarter (23.2%) in the moderate and approximately one in eight (16.2%) of those in the lower impairment groups reported high levels of suicidal ideation (H(2) = 73.66, *p* < = .001). The univariate odds ratio comparing the high impairment versus the lower impairment groups on this item was 2.39 [95% CI 1.94–2.93].

In summary, there was a clear gradient on virtually all relevant indices across the three functional impairment groupings. For example, for CMDs, persons with one or more disorders comprised one third (176, 31%) of the lower impairment group, a half (116, 53%) of the moderate impairment group, and three fourths (122, 74%) of the severe impairment group. The only index that did not conform to this pattern was that of physical ill-health (across all three groups, reporting of physical ill-health ranged from 71–77%).

As an indication of the convergence of psychosocial, mental and physical problems, those recording both a CMD and fair/poor physical health (580, 60%) reported significantly greater exposure to TEs (*m* = 12.9, sd = 7.04 vs m = 9.93, sd = 6.08; t(902) = −6.96, *p* < 0.001), peri-migration stressors (m = 8·26, sd = 3·22 m = 7·1, sd = 3·45; t(790) = −5.27, *p* < 0.001) and PMLDs (*m* = 14.44, sd = 5.15 m = 12.18, sd = 5.48; t(793) = −6.41; *p* < 0.001).

## Discussion

Our study revealed high levels of exposure to TEs, peri-migration stressors and PMLDs, amongst Rohingya refugees in general. Those stratified to the top twenty percent of functional impairment on the WHODAS reported high levels of exposure to all domains of psychosocial stressors as well as a high prevalence of CMDs, particularly the comorbid pattern. Importantly, one in two refugees in the high functional impairment group reported suicidal ideation. Overall, these findings suggest that the WHODAS may be effective as a screen used to identify refugees in greatest need of psychosocial and mental health services^17,18^.

The study strengths include the probabilistic approach to sampling, the large sample size and the high response rate (83%). Restriction of the sample to one country and ethnic grouping limits the generalizability of our findings regarding the general utility of the WHODAS as a screening measure for refugees. Further studies in other refugee settings are needed to test the universal applicability of our findings. In each setting, the WHODAS will need to be culturally and linguistically adapted and field tested in order to re-calibrate the cut-off points. In general, although we followed a rigorous method for adapting and testing measures, the risk of transcultural error in measurement remains. Retrospective bias in recall of trauma events and peri-traumatic stressors may introduce inaccuracies in reporting. We note, however, that the traumas and stressors reported are consistent with the known history of Rohingya refugees displaced throughout Southeast Asia^19^. Although we restricted the analysis to four CMDs, these diagnoses reflect the most general forms of mental disorder identified amongst refugees. We note that, contrary to our predictions, variations in reported physical ill-health showed little relationship to WHODAS stratification. It is possible that the measure used was too sensitive, failing to discriminate between severe and more moderate levels of physical ill-health. More detailed measures of physical ill-health, ideally corroborated by objective evidence, may improve the specificity of this index. Finally, our specification of a 20% threshold on the WHODAS was somewhat arbitrary and hence should be regarded as indicative only. We selected that threshold on the assumption that approximately half of the persons identified would ultimately seek and receive treatment, representing an attendance rate of 10% of the total refugee population (at a country level, approximately 5,000 persons of Rohingya background, excluding children and adolescents). Even that number of presentations to clinics would represent a major challenge in a setting such as Malaysia where there is a severe shortage of agencies specifically providing for the mental health of refugees. At the same time, it is likely that our estimates of those who would seek MHPSS services may be inflated given the cultural and practical barriers to seeking MHPSS assistance amongst the Rohingya. Factors that could inhibit seeking help include shame, social stigma, language issues, and concerns about confidentiality and impact of being seen to be mentally ill on rights to residency^1^.

Notwithstanding these limitations, our findings are of interest for several reasons. They offer the first systematic record of the extensive TEs, peri-migration stressors and PMLDs experienced by displaced Rohingya, the largest population of refugees currently dispersed across Southeast Asia^20,21^. Of particular note are the high rates of torture, imprisonment, and exposure to atrocities and murders reported by the sample. Further, the data reveal the extreme conditions that Rohingya experienced while traveling by boat to Malaysia, including extortion, extreme deprivations, and physical threats. In that respect, our study confirms other accounts of human rights agencies and UN missions detailing the extensive exposure to persecution and social duress experienced by the Rohingya population in general^22,23^. Traveling to Malaysia by boat (rather than over land) is an indicator of the urgency of the need to flee given the dangers involved in embarking on sea voyages in unsafe craft. In Malaysia, Rohingya refugees continue to experience severe challenges relating to inadequate housing, access to education and health, poverty, and in obtaining legal support. Our findings also detail the extent of the mental health problems experienced by these refugees, including the high prevalence of comorbid patterns of CMDs.

A key aim of the study was to assess whether a brief measure of functional impairment was able to identify a subpopulation of refugees exhibiting the psychosocial and psychiatric characteristics of persons who typically attend mental health services when they are available. The indicative approach of stratifying refugees into the top twenty percent stratum of functional impairment according to the WHODAS defined a subpopulation characterized by high levels of exposure to psychosocial stressors and in whom there was a concentration of comorbid CMDs, especially compared to the lower impairment group. At the same time, a substantial number of persons experiencing CMDs fell into the moderate and even lower functional impairment categories. That observation suggests that the presence of CMD on its own does not necessarily imply high levels of functional impairment, that is, many refugees continue to function and cope in spite of experiencing substantial levels of symptoms, for example, of depression^24^. Ideally, however, even these persons should be provided with mental health care if they so choose. The finding of severe impairment being associated with employment may be attributable to the poor working conditions confronted by Rohingya refugees in Malaysia.

In summary, our findings offer preliminary evidence that a brief screening measure of functional impairment, the WHODAS may prove useful in identifying refugees in greatest need of MHPSS attention. The data indicate a close convergence between high scores on the WHODAS and a constellation of MHPSS characteristics typical of persons attending mental health services, that is, exhibiting a combination of CMDs (including comorbidity) and severe background psychosocial stressors extending from exposure to trauma in the home country through to severe adversity in the peri- and postmigration environment. The urgency of need is further illustrated by the finding that half of those in the high functional impairment group experienced high levels of suicidal ideation. Further evaluations are needed to assess the utility of the WHODAS as a potential screening tool for MHPSS needs across a range of refugee groups from a range of cultures and backgrounds.

## Supplementary information

SUPPLEMENTARY FILE 1.

SUPPLEMENTARY FILE 2.
