# Supplementary material for: Functional impairment as a proxy measure indicating high rates of trauma exposure, post-migration living difficulties, common mental disorders, and poor health amongst Rohingya refugees in Malaysia
Source: Transl Psychiatry. 2019 Sep 2;9:213. doi: 10.1038/s41398-019-0537-z (PMC6718407; doi:10.1038/s41398-019-0537-z)
Supplement: Supplementary file 1 [file 41398_2019_537_MOESM1_ESM.docx]

**Supplementary File 1**

**A long history of persecution of Rohingya**

The Rohingya long represented the majority ethnic group in Maungdaw and Buthidaung, the only townships in Myanmar with a majority Muslim population. The 1982 Citizenship Act excluded the Rohingya from the list of officially recognized ethnic minorities and denied them many basic rights including citizenship, freedom of movement, access to healthcare and education, marital registration and the vote (1). This law effectively rendered the Rohingya the largest stateless group in the world. In recent years, religious antagonisms have led to systematic campaigns of violence and discrimination against the Rohingya (2, 3), resulting in mass displacements to neighbouring countries in Southeast and South Asia. Over the years, many Rohingya have fled to neighbouring countries including Bangladesh and Malaysia. A substantial number have also sought refuge in Saudi Arabia, Pakistan, India, with small numbers found in Nepal, Thailand and Indonesia (4). None of these countries is party to the 1951 Refugee Convention or 1967 Protocol, which poses challenges to efforts to provide international protection for Rohingya refugees. A minority of Rohingya have been resettled in high-income countries such as the United States, Canada, United Kingdom and Australia (4).

**Rohingya refugees in Malaysia**

Currently, there are over 70,000 Rohingya registered with UNHCR Malaysia and an estimated 30,000-40,000 more who remain unregistered. Many first arrived by boat in Thailand through dangerous travels over the Andaman Sea and were then smuggled or trafficked into Malaysia (5, 6). They live throughout Peninsular Malaysia. All unauthorized foreigners, including Rohingya fleeing Myanmar, are considered ‘illegal’ or ‘prohibited’ immigrants under the Immigration Act and therefore face ongoing threats of arrest and indefinite detention in deplorable conditions (7, 8). Rohingya refugees in Malaysia live in overcrowded housing and under protracted conditions of statelessness with lack of access to educational opportunities, employment, and healthcare (9, 10). Some Rohingya have lived for decades in Malaysia and have been able to set up some forms of livelihoods and/or receive remittances from relatives who managed to get resettled to Australia, Canada, Denmark, or Sweden (11) but many continue to live in precarious economic situations (12).Those who have been detained in immigration detention are at risk of malnutrition, physical and mental abuse, assault, exploitation, extortion and indefinite detention (13, 14).

**Base population and sampling**

In the absence of census data, we drew on the United Nations High Commissioner on Refugees (UNHCR) data which estimated that in 2018, approximately 70,000 displaced Rohingya resided in Malaysia. According to informants, including community leaders, members of non-government organizations, and the UNHCR, seventy five percent of the Rohingya community resided in dense geographical clusters in and surrounding the two major cities of Kuala Lumpur in Selangor state and Ipoh in Perak state. Our informants noted that as a close-knit cultural group, it was rare for Rohingya to live outside these clusters.

Applying a probability proportional-to-size cluster sampling framework, we identified 18 villages in Selangor and 31 in Perak which together would generate approximately generated equal proportions of Rohingya from each site. We applied a census approach in which we identified all Rohingya in the selected catchment areas. We used a ‘street walk’ approach (commencing at one end and progressing house by house to the other) to map and record the household composition and the total number of eligible respondents in each village. Eligible persons were all self-identified Rohingya either originating from Myanmar or the offspring of at least one Rohingya parent. We continued recruitment until we reached our target sample size of 1400 (approximately 700 in each of the two states). Of the 1156 adults approached, 197 (17%) declined interview, almost all citing ‘being too busy’, yielding a response rate of 83%. Non-participants did not differ in key sociodemographic characteristics (mean age 28 years, sd=4.8, 60% men) from the participating sample.

**Survey instruments**

We used the Refugee Mental Health Assessment Package (R-MHAP) to assess most of the indices used in the study. The R-MHAP is a comprehensive psychiatric and psychosocial assessment battery designed to assess background trauma exposure, ongoing stressors and common mental disorders (CMDs) of relevance to refugees (Tay et al., 2015b). We used the *Ruáingga* language version of the R-MHAP following a standard process of translation and back-translation (15).

*Mental Health Indices*

The common mental disorders (CMDs) assessed included point prevalence posttraumatic stress disorder (PTSD), major depressive disorder (MDD), generalized anxiety disorder (GAD) and persistent complex bereavement disorder (PCBD), categories that are frequently identified amongst refugees (15). We applied DSM-5 (Diagnostic and Statistical Manual, the 5^th^ edition) criteria for assigning probable disorders according to the presence and duration of symptoms and associated indications of emotional distress and impairment. We apply the term ‘probable’ in assigning diagnoses given that the R-MHAP is implemented by trained field workers, not mental health professionals. To generate probable caseness, we applied a conservative estimate in which each symptom needed to be rated either “quite a lot” or “extremely”.

Details of the steps taken to modify the diagnostic measures to ensure their cultural, semantic and linguistic appropriateness are reported herein. Specifically, symptoms of each CMD were adapted to the culture based on consultations with members of the Rohingya community in individual interviews and focus groups recruited using purposive sampling to ensure representation of different genders, levels of education, and roles in the society. The aim was to refine the content and expression of items to ensure their congruence with Rohingya language and cultural understandings. Participants in focus groups endorsed the relevance of the listed items of each disorder, suggesting modifications and improvements in the Rohingya terms used to align with the relevant DSM-5 criteria.

For PTSD, the reported items include fear (*Dorr*), nightmares (*horáf-kuab*), flashbacks (*dhubasa-mont-foron),* hypervigilance (*béniyomor-touçob*), insomnia (homot-hóulol-oum), irritability (aroushja), numbness (Besút), shock (*Dilot-do-ron*); for MDD, the endorsed items include hopelessness (*Asha-bara/ Na-ommaid*), low mood (*Dilor-joshba-home*), isolation (*Judaiyee*), sadness (*Peré-chani/ Ou-chanti*), for GAD, restlessness (*Chóit-goron*), feeling anxious (*Ba-fa-seintaat*), nausea (*Waijshon*), feeling nervous (*Dordor-lage*), palpitation (*Khoilla-dudo-fani*), Pain in the body (*gaa-bij*), tension (*Dilor-shiac*), tremor (*Hofon*), fatigue (*oran-péreshan*); for PCBD, the reported items include grief (*Dilor-furani*), guilt ((*dili) dhush*), loneliness (*Thon-haryee*), loss of family (*Haráiyé*), sorrow (*Afsus*), and feeling stunned (*Besúth/behush*).

Data from the subsequent survey indicated high levels of internal reliability for items comprising PTSD (Cronbach’s α= .95), MDD ((Cronbach’s α= .91), GAD (Cronbach’s α= .87), and PCBD (Cronbach’s α= .95).

Functional impairment was assessed using the WHO Disability Assessment Schedule-Short Form (WHODAS 2.0) [33]. The WHODAS assesses functioning in six core life domains of cognition/communication, mobility, self-care, interpersonal interaction, life activities, and participation in society. The items are rated on a 5-point Likert scale of functional impairment (1=none; 2=mild; 3=moderate; 4=severe; 5=extreme). The WHODAS has been widely used across epidemiological surveys worldwide, yielding sound internal consistency (for example, a Cronbach α=0.87 for the entire scale) and test-retest reliability (producing intraclass correlation coefficients ranging from 0.69 to 0.89 across items). The measure has shown reasonable correspondence with locally developed measures of functioning at different sites, a test of its ecological validity (16, 17). To facilitate standardization of classification across future studies, we applied international cut-offs derived from the WHO cross-country survey (18). A score ranging between 0 and 18 (corresponding to the 90th population percentile based on published normative data) was assigned to a lower impairment group (573, 59.8%); 19 and 31 (95th percentile) to a moderate impairment group (220, 22.9%); and 32 and above (97th percentile) to the severe impairment group (166, 17.3%).

Physical health was assessed using a widely used item derived from the 12-item Short Form Health Survey (SF-12), a measure of physical wellbeing developed for the Medical Outcomes Study (MOS), a longitudinal study of patients with chronic diseases (19). The item has shown high levels of reliability and validity in relation to other measures of physical health both in cross-sectional and longitudinal studies (20). Those who rated their physical health as good or excellent were assigned a score of 1, whereas those who rated their health as fair or poor were given a score of 0.

We assessed frequency of lifetime exposure to pre-migration traumatic events (TE) using a pre-established R-MHAP inventory adapted to the context and history of the Rohingya based on information obtained from individual and focus group interviews. In past studies we have encountered difficulties in accurately dating events or enumerating the frequency of their occurrence in societies where numeracy and literacy are low and where dates are afforded little cultural salience. Inquiring whether a type of trauma has occurred or not proves to be a more reliable measure, producing a trauma event (TE) count that has a demonstrated legacy of predicting mental disorder (21). The list we compiled included exposure to war, torture, persecution, rape, murders, physical injuries, imprisonment, witnessing atrocities, and witnessing deaths of family members. The TE count ranged from 0 to 12. Based on the distribution of scores, we derived three ordinal categories of TE exposure (0—10 counts, 11—20 counts, 20 counts or more).

We applied the same procedure to compile a list of peri-migration stressors again based on informant interviews and focus groups. The list included severe stresses and threats encountered during flight to Malaysia, such as extortion by people smugglers, starvation, physical assault, sexual abuse, and witnessing deaths or suicide of others. We applied the same binary response scale (exposed or not) as for the TE count. Based on distributions, the peri-migration stressor count (range 0 to 13) was rendered into three ordinal categories: 0—5 counts, 6—10 counts, 11 counts or more.

An inventory of post-migration living difficulties (PMLDs) was based on the Humanitarian Emergency Settings Perceived Needs (HESPER) scale (Semrau et al., 2012) and assessed common stressors confronted by the Rohingya communities in Malaysia. We adapted the measure to the local culture and context following an extensive process of qualitative adaptation based on individual and focus group interviews. For ease of analysis and interpretation, we collapsed the four-response format into binary categories (0= not a problem or a bit of a problem; 1= a moderately or a very serious problem), generating a total PMLD count based on an addition of all endorsed items (range 1 to 25). To achieve even distributions across subgroups with varying levels of exposure, we derived three ordinal categories based on the total count variable: 0—10 counts, 11—20 counts, 20 counts or more.

**Field Team Training**

The field team consisted of five men and three women drawn from the Rohingya communities in Selangor and Perak. They received three weeks’ intensive training by bilingual Bangladeshi clinical psychologists in basic mental health concepts and diagnosis, interviewing techniques and ethics. The team then piloted the survey in the field, refining their approach based on supervision and feedback. The field team received weekly onsite and remote supervision via videoconferencing throughout the survey.

**Statistical Analysis**

The sample size recruited was based on an a priori estimate of predicted odds ratio for key variables (3.4 or greater). We calculated frequency of exposure to pre-migration TEs, peri-migration stressors and PMLDs. We estimated prevalence of probable diagnoses for each of the four CMDs (PTSD, MDD, GAD, and PCBD) based on DSM-5 criteria. We conducted univariate analyses to assess associations between predictor variables and our tripartite functional impairment categorization. Predictor variables included social and demographic characteristics, length of residency, residency status, travel mode, premigration TEs (0—10 counts, 11—20 counts, 20 counts or more), peri-migration stressors (0—5 counts, 6—10 counts, 11 counts or more), PMLDs (0—10 counts, 11—20 counts, 20 counts or more), CMD status (any disorder, one disorder, or two or more disorders), and self-rated physical health (poor/fair vs good/excellent health). We then included statistically significant bivariate predictor variables in multinomial logistic regressions to identify their relative contributions in differentiating functional impairment categories. Education and length of residency were excluded because they were not statistically significant in univariate comparisons.

Application of the variance inflation factor (VIF) of 3.3 or greater indicated covariance amongst several predictor variables. To address this issue statistically, we conducted three separate regression models each comprising a coherent set of variables: Regression 1. Demographic and migration variables (age, gender, marital status, residency status, travel mode); Regression 2. Trauma and stressor variables: mean TE score, peri-migration stressor count, and mean PMLD score; Regression 3. CMD status and physical health. We report odds ratios (ORs) and adjusted odds ratios (AORs) with 95% confidence intervals (CIs) in each instance.

*Missing data*

Our auditing process identified random missing data based on an electronic transfer error that could not be corrected post hoc. The missing data were restricted to demographic variables and ranged from 4% (gender, mode of travel) to 22% (employment, education, marital status). The missing data were automatically excluded using listwise deletion in the relevant analyses.

**Results**

**Sociodemographic characteristics**

Mean age was 28.3 (sd=9.03) years and three quarters of participants were men (n=743, 77.5%). More than half were single (n=538, 56.1%). Nearly half (n=466, 48.6) held UNHCR protection status, that is, they had already been endorsed as refugees. The majority (687, 71.6%) had arrived in Malaysia by boat and the average length of residency was four years (47 months, sd=43.68). Over half (538, 56.1%) were illiterate or had no formal education; a quarter (261, 27.2%) had completed primary education. A large number were employed or were electively involved in domestic duties (632, 65.95%) and 120 (12.5%) were unemployed.

**Exposure to pre-migration traumatic events, peri-migration stressors, and post-migration living difficulties**

Individual categories of traumas experienced by more than 60% of the sample included torture (775, 81%); witnessing rape or sexual violence involving family members or friends (771, 80%); witnessing murder of family and friends (660, 69%); and witnessing mass killings and other atrocities (581, 61%). Highly endorsed peri-migration stressors included being confined in an overcrowded cargo boat for weeks (923, 96%); being subjected to extortion by people smugglers (915, 95%); exposure to extreme lack of food or water (828 86.3%); and being held in captivity together with others (734, 76.5%). Highly endorsed PMLDs rated as serious/very serious problems included poverty (90%); lack of aid (97%); poor access to health care (82%); inadequate shelter (78%); difficulties caring for family members (88%); and lack of access to information (73%).

References

1. Human Rights Watch. Perilous Plight: Burma's Rohingya Take to the Seas. 2009.

2. Holliday I. Addressing Myanmar’s citizenship crisis. Journal of Contemporary Asia. 2014;44(3):404-21.

3. Wade F. Myanmar's Enemy Within: Buddhist Violence and the Making of a Muslim'other'. USA: Zed Books; 2017.

4. Tay AK, Islam R, Riley A, Welton-Mitchell C, Duchesne B, Waters V, et al. Culture, Context and Mental Health of Rohingya Refugees: A review for staff in mental health and psychosocial support programmes for Rohingya refugees. . Geneva, Switzerland United Nations High Commissioner for Refugees (UNHCR)

2018.

5. Tazreiter C, Pickering, S., Powell. R. . Rohingya women in Malaysia: decision-making and information sharing in the course of irregular migration. European University Institute, Robert Schuma Centre for Advanced Studies, 2017 Contract No.: EUI Working Paper RSCAS 2017/55.

6. United Nations High Commissioner for Refugees. Mixed Movements in South-East Asia 2016. UNHCR, 2017.

7. Wake C. ‘Turning a blind eye: The policy response to Rohingya refugees in Malaysia. London, United Kingdom: Humanitarian Policy Group, Overseas Development Institute, 2016.

8. Wake C, Cheung, T. ‘We want to live in dignity:’Livelihood strategies of Rohingya refugees in Malaysia: . London, United Kingdom: Humanitarian Policy Group, Overseas Development Institute, 2016.

9. Verghis S. Access of Chin and Rohingya refugees and asylumseekers to maternal health services in the Klang Valley: Monash University; 2013.

10. Wake C. Forced Migration, Urbanization and Health: Exploring Social Determinants of Health Among Refugee Women in Malaysia. University of Victoria, 2014.

11. Huennekes J. Emotional Remittances in the Transnational Lives of Rohingya Families Living in Malaysia. Journal of Refugee Studies. 2018:doi:10.1093/jrs/fey036.

12. Aziz A. Urban refugees in a graduated sovereignty: the experiences of the stateless Rohingya in the Klang Valley. Citizenship Studies. 2014;18(8):839-54.

13. International Rescue Committtee. In search of survival and sanctuary in the city: Kuala Lumpur, Malaysia. 2012.

14. Ananthalakshmi A. Exclusive: More than 100 die in Malaysian immigration detention camps in two years. Reuters World news, March 30, 20172017.

15. Tay AK, Rees S, Chen J, Kareth M, Mohsin M, Silove D. The Refugee-Mental Health Assessment Package (R-MHAP); rationale, development and first-stage testing amongst West Papuan refugees. International journal of mental health systems. 2015;9(1):1-13.

16. Schneider M, Baron E, Davies T, Bass J, Lund C. Making assessment locally relevant: measuring functioning for maternal depression in Khayelitsha, Cape Town. Soc Psychiatry Psychiatr Epidemiol. 2015;50(5):797-806.

17. Scorza P, Stevenson A, Canino G, Mushashi C, Kanyanganzi F, Munyanah M, et al. Validation of the “World Health Organization Disability Assessment Schedule for Children, WHODAS-Child” in Rwanda. PLoS One. 2013;8(3):e57725.

18. Ustun TB, Chatterji S, Kostanjsek N, Rehm J, Kennedy C, Epping-Jordan J, et al. Developing the World Health Organization Disability Assessment Schedule 2.0. Bull World Health Organ. 2010;88(11):815-23.

19. Ware Jr JE, Kosinski M, Bayliss MS, McHorney CA, Rogers WH, Raczek A. Comparison of methods for the scoring and statistical analysis of SF-36 health profile and summary measures: summary of results from the Medical Outcomes Study. Med Care. 1995;33(4 Suppl):AS264-79.

20. Mollica RF, Sarajlic N, Chernoff M, Lavelle J, Vukovic IS, Massagli MP. Longitudinal study of psychiatric symptoms, disability, mortality, and emigration among Bosnian refugees. JAMA. 2001;286(5):546-54.

21. Steel Z, Chey T, Silove D, Marnane C, Bryant RA, Van Ommeren M. Association of torture and other potentially traumatic events with mental health outcomes among populations exposed to mass conflict and displacement: A systematic review and meta-analysis. JAMA - Journal of the American Medical Association. 2009;302(5):537-49.
