# Supplementary material for: Functional impairment as a proxy measure indicating high rates of trauma exposure, post-migration living difficulties, common mental disorders, and poor health amongst Rohingya refugees in Malaysia
Source: Transl Psychiatry. 2019 Sep 2;9:213. doi: 10.1038/s41398-019-0537-z (PMC6718407; doi:10.1038/s41398-019-0537-z)
Supplement: Supplementary file 2 [file 41398_2019_537_MOESM2_ESM.docx]

**Supplemental Table File 2**

**Supplementary File 2a: Exposure to premigration Traumatic Events (TEs) amongst the Rohingya refugees living in Malaysia (n=959)**

| **TEs experienced in Rakhine reported by the Rohingya refugees** | **N** | **%** |
| --- | --- | --- |
| Torture | 775 | 81% |
| Witnessing rape or sexual violence | 771 | 80% |
| No food or water | 660 | 69% |
| Witnessed murders of friends or family members | 611 | 64% |
| No shelter | 593 | 62% |
| Witnessing mass killings or atrocities (such as adults and children being burnt alive, disembowelment) | 581 | 61% |
| Forced to flee home to seek refugee elsewhere | 533 | 56% |
| Physical injuries during war | 382 | 40% |
| Home intentionally burnt down | 346 | 36% |
| Imprisonment | 330 | 34% |
| Family members taken away | 311 | 32% |
| Separated from family who had been persecuted | 248 | 26% |
| Witnessing murders of strangers | 244 | 25% |
| Injured while in combat | 111 | 12% |
| Fought in combat | 82 | 9% |

**Supplementary File 2b: Exposure to peri-migration stressors reported by the Rohingya refugees during flight to Malaysia (n=959)**

| **Extreme stressors reported during flight to Malaysia** | **N** | **%** |
| --- | --- | --- |
| Confined to overcrowded space or cargo with others for weeks | 923 | 96·2% |
| Extorted financially by people smugglers | 915 | 95·4% |
| Forced to hide in forests to escape police authorities | 872 | 90·9% |
| Had no food and water while traveling | 828 | 86·3% |
| Encountered a serious danger (e.g. boat capsized, drowning) | 775 | 80·8% |
| Held in captive or caged during voyage to Malaysia | 734 | 76·5% |
| Being physically unwell or family being physically unwell | 682 | 71·1% |
| Physically threatened, attacked, or injured during voyage to Malaysia | 583 | 60·8% |
| Forced to abandon people during journey to Malaysia | 571 | 59·5% |
| Witnessing deaths of others who committed suicide during journey to Malaysia | 545 | 56·8% |
| Witnessing murders of others (such as people being buried alive) by people smugglers | 534 | 55·7% |
| Witnessing rape or sexual violence involving women and children during journey to Malaysia | 274 | 28·6% |
| Separated from family during journey to Malaysia | 184 | 19·2% |

**Supplementary File 2c: Exposure to Postmigration Living Difficulties (PMLDs) amongst the Rohingya refugees living in Malaysia (n=959)**

| **Post-migration Living Difficulties** | **N** | **%** | **N** | **%** |
| --- | --- | --- | --- | --- |
|  | **Moderately serious problem** | | **Very serious problem** | |
| Income | 480 | 50·1% | 384 | 40·0% |
| The way aid is provided by any organizations | 444 | 46·3% | 488 | 50·9% |
| Access to healthcare | 418 | 43·6% | 370 | 38·6% |
| Shortage of shelter | 414 | 43·2% | 333 | 34·7% |
| Care for family members | 407 | 42·4% | 437 | 45·6% |
| Access to information | 376 | 39·2% | 328 | 34·2% |
| Support from others (e.g. relatives, neighbors) | 354 | 36·9% | 620 | 64·7% |
| Moving between places with limited transport | 351 | 36·6% | 585 | 61·0% |
| Safety in the community | 344 | 35·9% | 422 | 44·0% |
| Physical health | 316 | 33·0% | 72 | 7·5% |
| Discrimination | 305 | 31·8% | 253 | 26·4% |
| Stress | 303 | 31·6% | 182 | 19·0% |
| Shortage of food | 267 | 27·8% | 77 | 8·0% |
| Access clothes, bedding, or blankets | 239 | 24·9% | 91 | 9·5% |
| Separated from family | 205 | 21·4% | 488 | 50·9% |
| Being displaced from home | 172 | 17·9% | 275 | 28·7% |
| Law and justice in the community | 160 | 16·7% | 431 | 44·9% |
| Safety or protection for women from violence in the community | 152 | 15·8% | 178 | 18·6% |
| Shortage of water | 151 | 15·7% | 61 | 6·4% |
| Access to toilets | 144 | 15·0% | 82 | 8·6% |
| Being humiliated or treated badly by others | 137 | 14·3% | 102 | 10·6% |
| Personal hygiene | 132 | 13·8% | 31 | 3·2% |
| Access to education for children | 87 | 9·1% | 143 | 14·9% |
| Mental illness in the community | 30 | 3·1% | 35 | 3·6% |
| Substance use in the community | 8 | 0·8% | 5 | 0·5% |
